# Placebo-controlled clinical trials: how trial documents justify the use of randomisation and placebo

**DOI:** 10.1186/1472-6939-16-2

**Published:** 2015-01-11

**Authors:** Tapani Keränen, Arja Halkoaho, Emmi Itkonen, Anna-Maija Pietilä

**Affiliations:** Science Service Center, Kuopio University Hospital, P.O. Box 100, Kuopio, 70029 KYS Finland; Department of Pharmacy, University of Eastern Finland, P.O. Box 1627, 70211 Kuopio, Finland; National Institute for Health and Welfare, Mannerheimintie 170, P.O. Box 30, 00271 Helsinki, Finland; Department of Nursing, University of Eastern Finland, P.O. Box 1627, 70211 Kuopio, Finland

**Keywords:** Clinical trials, Placebo, Randomisation, Ethics, Justification

## Abstract

**Background:**

Randomised clinical trials (RCTs) involve procedures such as randomisation, blinding, and placebo use, which are not part of standard medical care. Patients asked to participate in RCTs often experience difficulties in understanding the meaning of these and their justification.

**Methods:**

We reviewed RCT protocols, statements of the principal investigator (PI), and participant-information materials, as submitted for opinion to a research ethics committee. We evaluated how the justification for the use of placebo was described in these documents and how the participants had been informed about randomisation, placebo use, and the possible risks of receiving placebo.

**Results:**

In total, 52 RCTs were identified. Eighteen of the study protocols (35%) provided some rationale for the use of placebo. In 15 (29%) of the statements, the PI had provided justification for its use. Possible risks related to placebo use were described in nine (17%) of the statements. An explanation as to why placebo was necessary featured in only 12 (23%) of the sets of participant-information materials, and only six (12%) of the documents discussed the possible risks associated with placebo.

**Conclusions:**

The justification of placebo control was inadequately described in the RCT study protocols, by principal or national co-ordinating investigators, and in participant-information documents. Furthermore, possible health-related risks associated with the use of placebo were poorly explained in the participant-information documents. Ethics committes and study participants need to be better informed of the rationale for the use of placebo, along with the associated risks.

## Background

Randomised clinical trials (RCTs) are the gold standard in the demonstration of efficacy and safety of new medical treatments. These trials involve certain procedures – such as randomisation, blinding, and the use of placebo – that are not part of standard medical care. Patients asked to participate in RCTs often have difficulties in understanding the meaning of these concepts and the justification for the procedures [1–4].

The ethics of placebo-controlled trials continue to be subject to heated debate [5, 6]. It is agreed that the use of placebo is acceptable when there is no proven intervention for the condition under study [6, 7]. In contrast, there is disagreement about whether placebo-controlled trials are ethically justifiable when effective treatment is available for the disorder in question [5, 8]. Placebo control may be permissible also in connection with disorders that already have a known effective treatment, when there are compelling and scientifically sound methodological reasons for its use and the participants in the study will not face additional risks of serious or irreversible harm from exposure to placebo [6, 7]. However, as Millum and Grady [6] point out, that placebo demonstrates the efficacy level of a new treatment may not be sufficient to justify its use.

It is important that patients recruited to RCTs understand the study methodology, the interventions to be used, and their benefits and risks. At present, little is known about how participants are informed of the placebo and its effects [9]. A key aim of the study described here were to evaluate how well the justification for the use of placebo had been described in the RCT protocols and by the principal/national co-ordinating investigators. Another aim was to determine the extent to which the participants had been informed about randomisation, placebo use, and any possible risks associated with placebo treatment. The analysis was based on review of study protocols, statements of the national and principal investigators (PIs), and information documents provided to the participants.

## Methods

We surveyed the records of the Research Ethics Committee of the North Savo Hospital District for 1 January 2006 to 31 December 2012 to identify all applications for randomised placebo-controlled clinical drug trials. This body is a local research ethics committee (REC) assessing study plans that encompass medical research on humans within the catchment area of the Kuopio University Hospital and, from October 2010, also the regions of the North Carelia Central Hospital, Mikkeli Central Hospital, and Savonlinna Central Hospital. A clinical trial was defined as a prospective study with human subjects that is intended to assess the efficacy, safety, or pharmacokinetics (absorption, distribution, metabolism, and excretion) of one or more medicinal products. Both industry-sponsored and investigator-initiated studies were included. Access to the files of the ethics committee was granted by the research director of the North Savo Hospital District.

### Data collection and analysis

Data were manually collected by two of the authors, but the entire multidisciplinary research group discussed and evaluated the studies selected. After identification of applicable studies, two of the authors, each independently, reviewed the protocols and their amendments, the ethics statements of the principal/national co-ordinating investigator, and participant-information documents. According to the national guidelines issued by the Finnish Medicines Agency [10] and the National Medical Research Ethics Committee [11], the principal or co-ordinating investigator for a clinical trial must deliver a statement on the ethical aspects of his or her study, which must address issues such as assessment of the rationale for the study, the risk/benefit ratio, and how informed consent is to be obtained.

Two of the authors recorded how the use of placebo had been justified in the study protocols, in the statement of the principal/national co-ordinating investigator, and in the participant-information documents. Furthermore, we searched for information in the statements of the co-ordinating investigators and in the participant-information documents about the following topics: randomisation, definition of ‘placebo’, and assessment of possible risks associated with the use of placebo.

Data were analysed by means of qualitative and quantitative content analysis in accordance with the research objectives via classification and grouping of quotes from the statements. The data were read through several times, with an overview created of the content. Meaningful combinations of words and sentences were extracted, condensed, and coded. After this, codes were grouped into two main categories: 1) justification for the use of placebo and 2) blinding and randomisation as described in the patient-information documents [12]. The entire analysis process was completed by two of the authors, independently. These two authors then discussed the findings, to reach agreement as to their opinions. After this, the findings were discussed by the whole research group, for unanimity and verified validity.

Also, quotes characterising the statements and the information were gathered and presented to illustrate the information provided to the ethics committee and to the people being asked to participate in the trials. Minor changes to the wording of the quotes were made for purposes of maintaining confidentiality.

## Results

### Description of the trials

In all, 52 RCTs were identified, mostly Phase III international multi-centre trials. The characteristics of the trials are presented in Table 1. The category ‘Others’ included disorders such as arthritis, osteoporosis, cardiovascular diseases, and sepsis.

**Table 1:** **Characteristics of the 52 randomised placebo-controlled clinical trials**

		***N***(%)
Phase of the clinical trial		
	II	11 (21)
	III	40 (77)
	IV	1 (2)
Type of trial		
	International multi-centre trial	46 (88)
	Investigator-initiated national study	6 (12)
Target condition of the trial		
	Depression	12 (23)
	Other psychiatric condition	4 (8)
	Diabetes	7 (13)
	Epilepsy	6 (12)
	Alzheimer’s disease and/or other cognitive disorders	6 (12)
	Cancer	1 (2)
	Obesity	2 (4)
	Others	14 (27)

In 42 of the trials, placebo was to be used in relation to disorders for which a standard therapy was available, and in nearly all of those cases (41 studies), standard therapy was expected to continue during the trial. Eleven of the 52 trials were connected with disorders for which there was no standard therapy available. Seven of the trials involved vulnerable populations – e.g., children or subjects with dementia.

### Justification for the use of placebo

Eighteen of the study protocols (35%) stated a rationale for the use of placebo. In most of these cases (12 protocols), the scientific rationale was presented as the justification, and six protocols cited regulatory guidelines as the reason for the use of placebo.

For all but one application, the statement of the principal/national co-ordinating investigator was available for evaluation. Fifteen (29%) of the PI statements presented some rationale for the use of placebo, and in all of these cases, scientific ends – i.e., confirming the efficacy of the study drug or a high placebo response rate for the disorder under study – were cited as the justification (e.g., ‘Placebo is used to scientifically confirm the efficacy of the study drug’). The investigators also explained that the use of placebo was acceptable because the participants would continue to receive the best available treatment during the trial. Possible risks related to placebo use were commented upon in nine (17%) of the statements. In all but one case, the investigator stated that there were no special risks associated with placebo.

The justification for placebo use was presented in only 12 (23%) of the participant-information documents. These represent the most frequently occurring phrasings: ‘Placebo is used in order to objectively assess the effects of study treatments’ and ‘The use of placebo as the comparator is justified because the subjects are already on the best available treatment’. None of the information documents provided any data on possible beneficial effects of placebo. Six (12%) of the information documents discussed possible risks associated with placebo by mentioning that the condition of the participants probably would not change, another stated that symptoms might worsen or remain unchanged. In addition, adverse effects of placebo were described as being comparable with the situation wherein no drug treatment is given.

The meaning of the term ‘placebo’ was explained in 36 (69%) sets of patient-information documents thus: ‘The placebo looks identical to the active study drugs but does not include any effective ingredients’. In all, 40 (77%) of the patient-information documents described the probability of receiving placebo via a statement that the subjects will have a 50% chance of receiving the study drug and a 50% chance of getting the placebo, while one statement explained: ‘The odds of receiving the placebo are one to three’.

### Blinding and randomisation as described in the patient-information documents

All sets of participant-information documents declared that the study had a double-blind design, described most often in the following manner: ‘This is a double-blind study, which means that neither you nor the study physician will know which of the study treatments you will receive’. Information related to possible emergency situations was given thus: ‘You, your doctor, and the study personnel will not be told which of the study drugs you will receive. However, in the event of an emergency, your doctor will be able to obtain the information on your treatment if this is necessary for your care’.

In total, 46 information documents (88%) described what the randomisation procedure meant in practice. The most common characterisation of randomisation was this: ‘As if by flipping a coin, a computer-based lottery or otherwise by chance’. However, in none of the cases was any justification for this procedure provided.

## Discussion

We analysed a sample of clinical trial protocols, ethics statements of principal/national co-ordinating investigators, and participant-information documents to assess how the use of randomisation and placebo had been justified and how possible risks associated with placebo use had been described. The main finding of our study is that all of these documents seem to overlook the information needs of the various stakeholders in clinical trials, especially those of the trial participants.

The main goal of clinical trial protocols is to present the aims, methods, and procedures of a scientific trial to investigators as well as to regulatory authorities. However, also RECs and funding agencies need to be able to assess the key trial elements, and they may not always have sufficient expertise to assess the adequacy of the study methodology. Therefore, study protocols should provide explicit justification for the choice of methods, such as the decision to use placebo control [8]. Only 35% of the protocols that we analysed presented any justification for the use of placebo, and regulatory requirements were stated to be the reason in most cases. According to the European Medicines Agency (EMA), the most commonplace primary objectives for pivotal clinical trials are to demonstrate superiority to placebo and to demonstrate non-inferiority or equivalence to an active control [13]. However, the agency’s guidelines recognise that a placebo control may sometimes not be suitable to address all study hypotheses. Therefore, for evaluation of a trial’s ethics, the protocol should discuss the feasibility and ethical acceptability of the use of placebo.

There are limited and partly contradictory published data on investigators’ attitudes and opinions on how participants should be informed about trials. In a survey of oncologists, the investigators considered it to be very important that participants understand the nature of trials, alongside the role of randomisation and placebos [14]. In another study, most of the investigators were confident that they had given enough information to their patients, and they expressed the belief that patients generally comprehend the implications of participation [15]. On the other hand, a study with 170 breast-cancer specialists found that only 12% of the physicians deemed the patients able to understand the information they needed in order to give informed consent. Hereu et al. [16] reported that a sample of investigators considered treatment allocation and the use of placebo among the least important elements in informing patients of clinical trials. The statements of principal/national co-ordinating investigators analysed in our study provided some justification for the use of placebo in only about a third of the cases, and under 20% of the statements discussed any possible risks related to placebo. The statements may not, however, reflect the attitudes of the investigators toward informing patients so much as those toward the REC. There are studies reporting that investigators do have concerns about patients’ best interests and possible study-related harm [17, 18].

It has been claimed that, while many clinical trial participants are satisfied with the information they have received and report that they understand the trial design, a substantial proportion of the subjects have poor or only partial comprehension of the goals and methods of a trial [16, 19–22]. The concept of randomisation seems to remain difficult to conceptualise [1–4]. Furthermore, some who understand what randomisation means have been found to be reluctant to accept its use [4], though this finding has not been confirmed in other studies [23, 24]. In our sample of studies, most of the participant-information documents described randomisation with brief, simple wording that has been found to be comprehensible to patients [25]. However, none of the participant-information documents provided any explanation as to why the randomisation procedure was necessary. Because patients tend to think that treatment allocation should be determined by clinical and personal characteristics [2], lack of any explanation for the use of randomisation may lead to confusion and even refusal to participate. On the other hand, if patients are not informed that clinical equipoise between the treatments compared in a trial is a fundamental requisite, they may think that randomisation is a lottery [3].

The probability of receiving one of the treatments (including placebo) was stated in about 70% of the cases we examined. Whilst in the survey of Bishop et al. [9], the chances of receiving a placebo were reported upon in all the information documents, the people being asked to participate in nearly one third of our cases were not able to make a truly informed decision on participation, on account of lack of knowledge of the probability of receiving active vs. placebo treatment. They were also unable to understand the possible benefits and harm associated with the study fully.

Those asked to participate in the trials were clearly informed of the possibility of receiving a placebo. The placebo treatment was described as a product that looks similar to the active study drug but is pharmacologically inert. This is consistent with Bishop et al’.s findings. In a large majority (77%) of the participant-information documents, no rationale for the use of placebo was presented. This is in quite striking contrast to the findings of Bishop and colleagues [9], who found an explanation for the use of placebo in 78% of the participant-information documents they analysed. The difference may be due to variations among national guidelines for providing participant information and to the diversity of funding sources and trial types. In a parallel with the findings of Bishop et al. [9], scientific reasons were given as the justification for the use of placebo in many information documents evaluated in our study. In only a minority of cases (12%) were the subjects informed about the possible risks of receiving the placebo. In the study of Bishop et al. [9], possible adverse effects related to the use of placebo were mentioned in 49% of the information documents. Bishop and colleagues concluded that the information documents they examined encouraged participants to focus on the target treatment. The use of placebo does not automatically imply that the subjects remain without any treatment. As in the series of studies surveyed here, in most trials the participants continued to receive standard care, with the study drug added to the existing treatment. It is possible that subjects receiving placebo may, however, experience a sub-optimal response to their concurrent medication.

The limitations of our study include its small sample size. Furthermore, the studies analysed involve mainly trials related to psychiatric and neurological disorders, while diabetes, cancer, and cardiovascular disorders represented only a minority. On the other hand, we were able to survey all the studies submitted for ethics committee evaluation over a reasonable span of time. We are aware of only one previous study analysing how placebo had been described in clinical trials’ participant-information documents [9]. In that survey, most of the studies analysed were non-commercial, whereas nearly all the studies in our sample were sponsored by a pharmaceutical company. In addition to participant-information documents, we were able to analyse the study protocols and investigator statements.

## Conclusions

Ethicists have proposed that RECs should demand that sponsors and investigators justify the use of placebo controls in all studies, especially whenever the study may involve withdrawal or withholding of proven effective therapy [8]. Furthermore, RECs should approve studies only when sufficient information has been provided for a solid judgement about the acceptability or undesirability of a placebo control. Furthermore, it has been stated that patients should participate in such studies if they have given fully informed consent, in awareness of the random allocation, the double-blind conditions, and the use of placebo [26]. As is discussed above, subjects asked to participate in clinical trials may not have an appropriate understanding of the study methods and procedures or of the possible harm as opposed to simply the possible benefits. It is known that patients may underestimate the risks and discomfort of participation in clinical trials [21]. Our study shows that, at least with respect to the written information, the documents provided have many shortcomings in their description of issues important for ensuring real informed consent. We agree with the conclusions of Bishop et al. [9] that there is a clear ethical need for greater transparency and more respect for the participants in the provision of written information about placebos.
